# The value of tibial mounted inertial measurement units to quantify running kinetics in elite football (soccer) players. A reliability and agreement study using a research orientated and a clinically orientated system

**DOI:** 10.1016/j.jelekin.2019.01.001

**Published:** 2019-02

**Authors:** Tom Hughes, Richard K. Jones, Chelsea Starbuck, Jamie C. Sergeant, Michael J. Callaghan

**Affiliations:** a Manchester United Football Club, AON Training Complex, Carrington, Manchester, UK; b Arthritis Research UK Centre for Epidemiology, Centre for Musculoskeletal Research, Manchester Academic Health Science Centre, University of Manchester, Manchester, UK; c Health Sciences Research Centre, School of Health Sciences, University of Salford, Salford, UK; d Centre for Biostatistics, University of Manchester, Manchester Academic Health Science Centre, Manchester, UK; e Department of Health Professions, Manchester Metropolitan University, Manchester, UK

**Keywords:** Accelerometer, Gait, Symmetry, Lower extremity

## Abstract

In elite football, measurement of running kinetics with inertial measurement units (IMUs) may be useful as a component of periodic health examination (PHE). This study determined the reliability of, and agreement between a research orientated IMU and clinically orientated IMU system for initial peak acceleration (IPA) and IPA symmetry index (SI) measurement during running in elite footballers. On consecutive days, 16 participants performed treadmill running at 14kmph and 18kmph. Both IMUs measured IPA and IPA SI concurrently. All measurements had good or excellent within-session reliability (intraclass correlation coefficient (ICC_2,1_) range = 0.79–0.96, IPA standard error of measurement (SEM) range = 0.19–0.62 g, IPA SI SEM range = 2.50–8.05%). Only the research orientated IMU demonstrated acceptable minimal detectable changes (MDCs) for IPA at 14kmph (range = 7.46–9.80%) and IPA SI at both speeds (range = 6.92–9.21%). Considering both systems, between-session IPA reliability ranged from fair to good (ICC_2,1_ range = 0.63–0.87, SEM range = 0.51–1.10 g) and poor to fair for IPA SI (ICC_2,1_ range = 0.32–0.65, SEM range = 8.07–11.18%). All MDCs were >10%. For IPA and SI, the 95% levels of agreement indicated poor between system agreement. Therefore, the use of IMUs to evaluate treadmill running kinetics cannot be recommended in this population as a PHE test to identify prognostic factors for injuries or for rehabilitation purposes.

## 1 Introduction

Periodic health examination (PHE) is a core component of healthcare practice in professional football (soccer) (Hughes et al., 2018) and is used by 94% of elite teams (McCall et al., 2016). The purposes of PHE are to identify prevalent conditions that may be a threat to safe participation (Ljungqvist et al., 2009), to monitor performance (Whatman et al., 2011) or rehabilitation progress (Hughes et al., 2018), and to identify potential prognostic factors (predictors) for injuries (Hughes et al., 2017). This is achieved using general medical tests, musculoskeletal examination techniques (Ljungqvist et al., 2009) and evaluation of functional performance (Hegedus et al., 2015, Whittaker et al., 2017). Running is a key functional component of elite football (Bangsbo et al., 2006), so analysis of running kinetics may be useful as a PHE tool, although it has not yet been investigated in this population (Hughes et al., 2017).

During running, ground reaction forces (GRFs) occur within the first 50–60 ms of every heelstrike, which result in tibial shock and compressive loading of musculoskeletal structures (Lafortune and Hennig, 1991). The bilateral distribution of these forces can be quantified using the symmetry index (SI) (Robinson et al., 1987), expressed as a percentage difference between limbs, with a value of zero percent indicating perfect symmetry (Zifchock et al., 2008). It has been hypothesised that kinetic asymmetries may increase injury risk due to between-limb loading differences (Tenforde et al., 2018), although this is currently unsubstantiated. Indeed, some asymmetry appears to occur naturally at running speeds, where uninjured runners have shown asymmetry values of 3.1% for vertical GRF and 31.7% for tibial shock values (Zifchock et al., 2006). This may be due to limb dominance or differences in neuromuscular control mechanisms between legs (Sadhegi et al., 2000). Footballers perform repetitive kicking actions which place different demands on the support limb and kicking limb musculature (Brophy et al., 2007). In theory, these training adaptations could also affect the magnitude of running kinetic asymmetry in this population.

Running kinetics have been traditionally quantified with force platforms that measure external GRFs (Lafortune, 1991, Raper et al., 2018) or custom designed, lab-based accelerometers that measure internal tibial shock (Kavanagh and Menz, 2008). However, these technologies are expensive and limited to a laboratory setting, which may prohibit expression of natural running patterns (Charry et al., 2013) and restricts their clinical usefulness. Alternatively, commercial skin mounted inertial measurement units (IMUs) have been developed which are affordable and portable (Liikavainio et al., 2007), so can be used in various environments which may allow natural running to occur (Charry et al., 2013). IMUs contain accelerometer components which, if mounted to the skin overlying the tibia, quantify internal tibial shock (Lafortune et al., 1995) through measurement of axial tibial initial peak accelerations (IPAs) (Lafortune and Hennig, 1991, Liikavainio et al., 2007).

IMUs have been used extensively for research purposes (Bergamini et al., 2014, Dowling et al., 2011). Research orientated IMU systems typically require expertise in complex data processing (to select appropriate signal filtering) and analysis (Ferguson et al., 2015), thus allowing data handling flexibility which can be tailored to the needs of the user or study objectives. Recently, clinically orientated IMU systems have been developed that use proprietary software algorithms (Kavanagh and Menz, 2008) to automate data processing and analysis, thereby eliminating the need for operator expertise (Wright et al., 2017). These systems also use relatively simple software interfaces to present a restricted set of metrics which are preselected by the manufacturer (Ferguson et al., 2015) and are representative of typical kinetic or kinematic data used to measure the performance of specific functional tasks (Al-Amri et al., 2018). Despite being less flexible, clinically orientated IMU systems offer a simple and time efficient method of objective kinetic running analysis (Charry et al., 2013) which is attractive to practitioners and may have considerable practical utility, especially during PHE.

IMUs should be accurate and reliable (Raper et al., 2018) if used for research or clinical purposes. IMUs should also be valid, but validation of skin mounted IMUs has been difficult because there is no established comparable technique to measure internal acceleration of body segments (Kavanagh and Menz, 2008). In the absence of a gold standard it is important to investigate the agreement between similarly established systems (Bland and Altman, 1986). Although there have been no studies that have investigated agreement between IMU systems, a research orientated IMU system has previously been shown to have good between-session reliability for tibial acceleration measurements at various running speeds when measured at 1 week and 6 months (Sheerin et al., 2018). However, as male recreational runners were investigated, it is unlikely that these results are generalisable to elite football players.

Therefore, the aims of this study were to determine the reliability of, and agreement between a research orientated and a clinically orientated IMU system for IPA and IPA SI measurement during running in elite footballers, to establish whether kinetic evaluation of running with IMUs could be acceptable for PHE or clinical use.

## 2 Materials and methods

This study was conducted and is reported in accordance with the Guidelines for Reporting Reliability and Agreement Studies (Kottner et al., 2011).

### 2.1 Participants

A convenience sample was selected from a cohort of elite football players under contract at an English Premier League Football Club. All data were captured from mandatory PHE processes completed through the participants’ employment. The anonymity and rights of all participants were protected. The football club granted permission to use these data. The use of the data for the current purpose was approved by the Research Ethics service at the University of Manchester.

### 2.2 Eligibility criteria

Participants were included if they (i) were >16 years and <40 years old; (ii) had trained fully without injury and were available for match selection within two weeks prior to testing. Participants were excluded if they (i) were a goalkeeper; (ii) had undergone previous major lower extremity joint surgery; (iii) suffered a systemic illness within the week before testing; (iv) had a leg length difference of >1 cm.

### 2.3 Preparation

Baseline measurements were recorded of (i) standing height (centimetres) and body mass (kilograms) using a height measure and scale (SECA 220, SECA, Hamburg, Germany), (ii) participants’ preference for kicking and non-kicking leg. True leg length measurements were recorded for each limb using a cloth tape measure, as described by Magee (2008). Throughout participation, all participants wore the same footwear and were required to use orthotics if previously prescribed by a podiatrist.

The IMUs were applied simultaneously according to the manufacturer’s instructions. The clinically orientated system used was ViPerform (Dorsavi, Melbourne, Australia) and the research orientated system was Delsys Trigno IM (Delsys Inc, Natick, Massachusetts, USA). Each ViPerform sensor consisted of a triaxial accelerometer which sampled at 100, 20 and 20 Hz on the x,  y and z axes, and correlated with vertical, anterior–posterior and medio-lateral directions respectively (Charry et al., 2013). A proprietary leg template based on each participant’s height was used to position the IMU. Disposable application pads (Dorsavi, Melbourne, Australia) were affixed to the medial tibia at the corresponding site and the IMUs were then clipped into position. ViPerform IMU application was completed by the same physiotherapist (TH) who was experienced with this system.

**Fig. 1:**
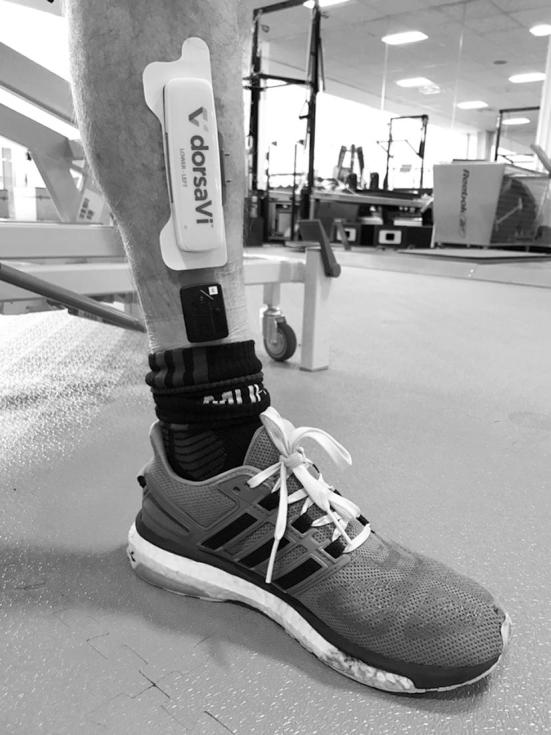
Photograph of inertial measurement unit placement on left tibia.

Each Delsys Trigno IM sensor contained an integrated tri-axial accelerometer which sampled at 148.1 Hz. The sensor was attached to Trigno Adhesive Skin Interfaces (Delsys Inc, Natick, Massachusetts, USA) and firmly sited in line with, but 2 cm distal to, the distal border of the ViPerform sensor (Fig. 1). This separation eliminated contact between units which could have distorted the acceleration data. The fixation of both IMUs was reinforced with a neoprene calf sleeve. Delsys IMU application was completed by either of two biomechanists (RKJ/CS), who were experienced with this system. The same examiner was used for the retest session.

### 2.4 Procedure

Testing sessions were performed before the participants commenced their daily training activities, 2 days following game participation to reduce potential fatigue effects. To warm up, participants cycled for 5 min on an exercise bicycle without resistance. Following the warm up, participants ran at 10kmph for 3 min on a treadmill (Woodway Desmo Pro, Waukesha, USA), to allow familiarisation and increase cardiovascular activity. A 60 s rest was provided before data collection. For the test protocol, to remove any effects of treadmill acceleration or deceleration on IPA data, the treadmill speed was pre-set and standardised to 14kmph. Participants ran at this speed for 60 s, followed by a 60 s rest period. This was repeated twice further, giving three repetitions in total. During the rest period following the third repetition, the treadmill speed was increased to 18kmph and a further three trials were completed with corresponding 60 s rest periods, after which data collection was concluded. Participants completed their usual training routine after the first testing session. The same protocol was repeated approximately 24 h following the first session to eliminate training exposure effects.

### 2.5 Data analysis

All ViPerform data were transferred wirelessly and processed in real time using the manufacturer’s software (ViPerform 5.10, Dorsavi, Melbourne, Australia) by the same person (TH). Delsys data were transferred wirelessly and retrospectively processed by the same biomechanist (CS). Custom written Matlab (Matlab, R2016A, MathsWorks, Natrick, MA, USA) code was used to process Delsys data with a 2-tap averaging filter, in order to achieve comparability between systems.

The amount of heelstrike impact shock was quantified using axial tibial IPA measurements from both systems, as described previously (Charry et al., 2013, Gill and O’Connor, 2003). IPA measured in gravitational force (g) was the primary parameter, collected separately for each limb. This was the average value of all IPA measurements recorded for each footstrike that occurred during a trial. These data were then used to calculate the SI as described by Robinson et al. (1987).

$$\mathrm{SI}\left(\%\right)=\frac{\mathrm{IPA}\;nonpref-IPA\;pref}{0.5\left(IPA\;nonpref+IPA\;pref\right)}\times 100$$

In this equation, *IPA nonpref* is the IPA measured for the non-preferred kicking leg and *IPA pref* is the IPA measured for the preferred kicking leg. $SI\left(\%\right)$ is the symmetry index expressed in percentage terms. A SI value of 0 indicates perfect symmetry, i.e. no difference in IPA between legs. Positive SI values indicate greater IPA magnitude on the non-preferred leg, whereas negative values indicate greater IPA magnitude on the preferred kicking leg.

Within-session analyses were conducted for IPA and IPA SI measurements, where data were compared across all 3 trials within each testing day and running speed, and stratified according to kicking limb preference. For between-session analyses, mean IPA and mean IPA SI values were calculated separately for each testing day, using data obtained for all 3 trials. These mean values were then compared across testing days for both running speeds and according to limb kicking preference. Within and between-session intraclass correlation coefficients (ICCs_2,1_) with corresponding 95% confidence intervals (CIs) were calculated and interpreted as: poor <0.40, fair = 0.40–0.70, good = 0.70–0.90 and excellent >0.90 (Coppieters et al., 2002). The standard error of measurements (SEM) and minimal detectable changes (MDC) were calculated for all measurements as described by Weir (2005). Because IPA was measured in gravitational force (g), IPA MDCs were also converted to percentage terms to assist clinical interpretation, using the formula presented by Flansbjer et al. (2005). For both IPA and IPA SI, MDCs were considered acceptable if they were <10%.

To determine agreement between systems for IPA and IPA SI, Bland–Altman plots and respective 95% limits of agreement (LOA) were produced (Bland and Altman, 1986), according to limb preference, running speed and testing day. Agreement was considered acceptable if the 95% LOA fell within a 1 g range for IPA, and 10% range for SI. Statistical analyses were completed using STATA 14 (StataCorp LLC, Texas, USA).

## 3 Results

### 3.1 Study participation and missing data

Sixteen individuals participated, where the mean (±standard deviation) characteristics of the sample were: age 17.36 (±1.25) years; height 180.21 (±6.41) cm; mass 74.15 (±5.33) kg. Orthotics were used in the training shoes of 3 participants for both testing sessions. The final sample size used in all analyses was n = 15, because a technical fault affected ViPerform IPA data from one participant on Day 1 and so they were excluded.

Within-session and between-session ICCs and 95% CIs, SEM and MDC values for IPA measurements are summarised in Tables 1 and 2 respectively. Descriptive, within-session and between-session reliability statistics for SI are presented in Table 3.

**Table 1:** Within-session descriptive statistics, ICC _2,1_ & 95% CIs, SEM and MDC statistics – Initial Peak Acceleration.

		Day 1										Day 2									
		Preferred leg					Non-preferred leg					Preferred leg					Non-preferred leg				
Speed	System	Mean g (SD)	ICC (95% CI)	ICC rating	SEM g	MDC g (%)	Mean g (SD)	ICC (95% CI)	ICC rating	SEM g	MDC g (%)	Mean g (SD)	ICC (95% CI)	ICC rating	SEM g	MDC g (%)	Mean g (SD)	ICC (95% CI)	ICC rating	SEM g	MDC g (%)
14kmph	ViPerform	6.98 (1.56)	0.89 (0.77–0.96)	Good	0.51	1.40 (20.11)	6.11 (0.96)	0.86 (0.71–0.95)	Good	0.36	0.99 (16.20)	6.76 (1.26)	0.91 (0.78–0.96)	Excel.	0.21	0.58 (8.65)	5.98 (0.69)	0.91 (0.81–0.97)	Excel.	0.29	0.80 (13.33)
	Delsys	7.79 (1.23)	0.96 (0.91–0.99)	Excel.	0.25	0.69 (8.80)	7.59 (1.14)	0.95 (0.89–0.98)	Excel.	0.25	0.69 (9.03)	7.91 (1.23)	0.95 (0.88–0.98)	Excel.	0.28	0.78 (9.80)	7.25 (0.96)	0.96 (0.91–0.98)	Excel.	0.19	0.54 (7.46)
18kmph	ViPerform	9.69 (1.98)	0.90 (0.79–0.96)	Excel.	0.62	1.72 (17.73)	8.44 (1.31)	0.86 (0.71–0.95)	Good	0.48	1.34 (15.92)	9.33 (1.68)	0.91 (0.80–0.97)	Excel.	0.51	1.40 (15.03)	8.36 (1.17)	0.83 (0.65–0.93)	Good	0.48	1.34 (16.08)
	Delsys	10.66 (1.35)	0.96 (0.89–0.99)	Excel.	0.27	0.74 (6.97)	10.47 (1.29)	0.96 (0.90–0.98)	Excel.	0.27	0.75 (7.17)	10.60 (1.44)	0.91 (0.80–0.97)	Excel.	0.44	1.21 (11.43)	9.97 (1.23)	0.86 (0.70–0.94)	Good	0.47	1.29 (12.95)

Key: IPA = initial peak acceleration; SD = standard deviation; ICC = intraclass correlation coefficient; CI = confidence interval; SEM = standard error of measurement; MDC = minimal detectable change; g = gravitational force; kmph = kilometres per hour; Excel. = excellent. Note: ICC ratings are as follows: poor <0.40, fair = 0.40–0.70, good = 0.70–0.90 and excellent = >0.90 (Coppieters et al., 2002).

**Table 2:** Between-session descriptive statistics, ICC _2,1_ & 95% CIs, SEM and MDC statistics – Initial Peak Acceleration.

		Preferred leg					Non-preferred leg				
Speed	System	Mean g (SD)	ICC (95% CI)	ICC rating	SEM g	MDC g (%)	Mean g (SD)	ICC (95% CI)	ICC rating	SEM g	MDC g (%)
14kmph	ViPerform	6.87 (1.38)	0.75 (0.40–0.91)	Good	0.70	1.93 (28.10)	6.04 (0.81)	0.76 (0.42–0.91)	Good	0.40	1.11 (18.28)
	Delsys	7.85 (1.22)	0.83 (0.56–0.94)	Good	0.51	1.41 (17.95)	7.42 (1.06)	0.83 (0.50–0.94)	Good	0.44	1.22 (16.49)
18kmph	ViPerform	9.51 (1.79)	0.63 (0.19–0.86)	Fair	1.10	3.04 (31.95)	8.40 (1.18)	0.87 (0.66–0.95)	Good	0.42	1.18 (14.01)
	Delsys	10.63 (1.37)	0.80 (0.51–0.93)	Good	0.61	1.68 (15.79)	10.22 (1.25)	0.68 (0.27–0.88)	Fair	0.70	1.95 (19.08)

Key: IPA = initial peak acceleration; SD = standard deviation; n = participants; ICC = intraclass correlation coefficient; CI = confidence interval; SEM = standard error of measurement; MDC = minimal detectable change; kmph = kilometres per hour; Excel. = excellent. Note: ICC ratings are as follows: poor <0.40, fair = 0.40–0.70, good = 0.70–0.90 and excellent = >0.90 (Coppieters et al., 2002).

**Table 3:** Within and between-session descriptive statistics, ICC _2,1_ & 95% CIs, SEM and MDC statistics – Symmetry Index.

		Day 1					Day 2					Between-session				
Speed	System	Mean % (SD)	ICC (95% CI)	ICC rating	SEM %	MDC %	Mean % (SD)	ICC (95% CI)	ICC rating	SEM %	MDC %	Mean % (SD)	ICC (95% CI)	ICC rating	SEM %	MDC %
14kmph	ViPerform	−12.03 (17.67)	0.79 (0.59–0.92)	Good	8.05	22.32	−10.97 (20.28)	0.94 (0.86–0.98)	Excel.	4.98	13.80	−11.50 (18.27)	0.63 (0.17–0.86)	Fair	11.18	30.99
	Delsys	−2.40 (10.01)	0.89 (0.76–0.96)	Good	3.32	9.21	−8.32 (14.17)	0.95 (0.88–0.98)	Excel.	3.24	8.99	−5.36 (12.39)	0.58 (0.13–0.83)	Fair	8.07	22.37
18kmph	ViPerform	−12.93 (16.53)	0.84 (0.68–0.94)	Good	6.51	18.04	−10.42 (17.51)	0.85 (0.69–0.94)	Good	6.79	18.81	−11.67 (16.21)	0.65 (0.22–0.87)	Fair	9.64	26.71
	Delsys	−1.62 (8.64)	0.92 (0.82–0.97)	Excel.	2.50	6.92	−5.89 (11.58)	0.94 (0.86–0.98)	Excel.	2.90	8.03	−3.75 (10.25)	0.32 (-0.14–0.71)	Poor	8.27	22.92

Key: SI = Symmetry index; SD = standard deviation; ICC = intraclass correlation coefficient; CI = confidence interval; SEM = standard error of measurement; MDC = minimal detectable change; kmph = kilometres per hour; Excel. = excellent. Note: −ve figures indicate greater IPA magnitude was on the preferred kicking leg; ICC ratings are as follows: poor <0.40, fair = 0.40–0.70, good = 0.70–0.90 and excellent = >0.90 (Coppieters et al., 2002).

### 3.2 Within-session reliability

For within-session IPA at both speeds, ViPerform IMU ICCs were good to excellent (range = 0.83–0.91) and Delsys IMU ICCS were excellent (0.91–0.96), with the exception of the non-preferred leg at 18kmph on Day 2 which had good reliability (ICC = 0.86). IPA SEMs were larger for the ViPerform IMU (0.21–0.62 g) compared with the Delsys IMU (0.19–0.47 g), with the exception of the preferred leg at 14kmph on Day 2. ViPerform MDC values of 0.58–1.72 g (8.65–20.11%) were larger than the 0.54–1.29 g (6.97–12.95%) values for the Delsys system, again with the exception of the preferred leg at 14kmph on Day 2. For IPA SI, ICCs for both systems were good to excellent. ViPerform IMU ICCs ranged between 0.79 and 0.94, while the Delsys system demonstrated consistently higher ICCs, between 0.89 and 0.95. The Delsys system demonstrated consistently lower SEM and MDC values; SEMs ranged between 4.98 and 8.05% for ViPerform and 2.50–3.32% for the Delsys IMU. MDCs ranged between 13.80 and 22.32% for ViPerform and 6.92–9.21% for the Delsys IMU.

### 3.3 Between-session reliability

For IPA, between-session reliability was good at 14kmph for both systems (ViPerform ICCs 0.75 and 0.76, Delsys ICC = 0.83 for both limbs). At 18 kmph, ICCs varied from fair to good between limbs for both systems (ViPerform ICCs 0.63 and 0.87, Delsys ICCs 0.68 and 0.80). On the preferred leg at both speeds, Delsys SEMs were 0.51 g and 0.61 g and MDCs were 1.41 g (17.95%) and 1.68 g (15.79%). These were generally less than for the ViPerform IMU (SEMs = 0.70 g and 1.10 g, MDC = 1.93 g (28.10%) and 3.04 g (31.95%). At both speeds and on the non-preferred leg, ViPerform SEMs were 0.40 g and 0.42 g, while MDCs were 1.11 g (18.28%) and 1.18 g (14.01%). In comparison, Delsys IMU SEMs were greater at 0.44 g and 0.70 g, while MDCs were also greater at 1.22 g (16.49%) and 1.95 g (19.08%).

For IPA SI, between-session ViPerform IMU reliability was fair across both speeds (ICC = 0.63 and 0.65) whereas Delsys IMU reliability was poor to fair (0.32 and 0.58). For both speeds, Delsys had smaller SEM (8.07% and 8.27%) and MDC values (22.37% and 22.92%) than the ViPerform (SEM = 9.64% and 11.18%, MDC = 26.71% and 30.99%).

### 3.4 Between-system agreement

**Fig. 2:**
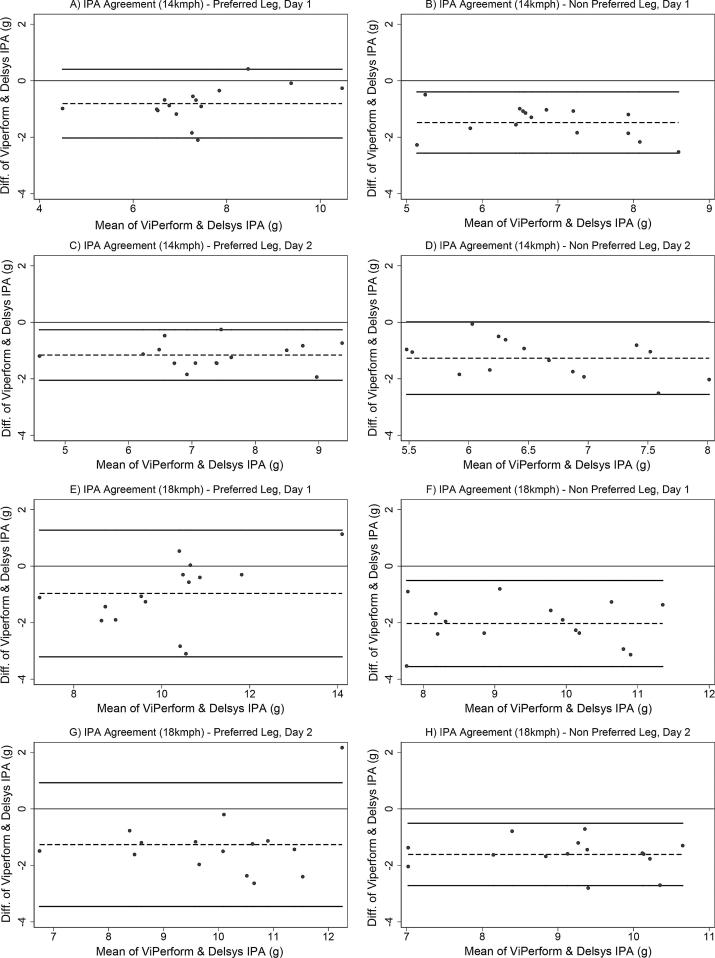
Bland-Altman plots to demonstrate agreement between systems for IPA measures at 14 and 18kmph on both testing days. Key: IPA = initial peak acceleration; diff = difference; g = gravitational force; kmph = kilometres per hour. Note: Thick black lines correspond to 95% limits of agreement, dashed black line corresponds to observed mean agreement. Where the y axis is 0, this indicates line of perfect agreement.

**Fig. 3:**
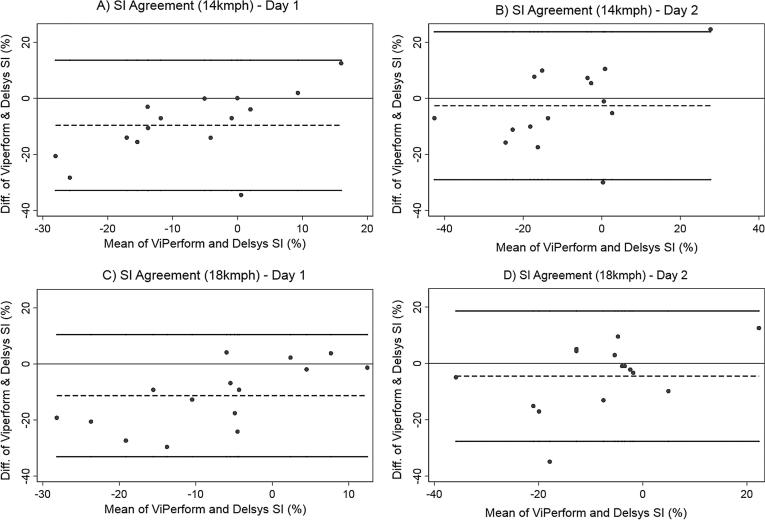
Bland-Altman plots to demonstrate agreement between systems for IPA SI on both testing days. Key: SI = symmetry index; diff = difference; % = percentage; kmph = kilometres per hour. Note: Thick black lines corresponds to 95% limits of agreement, dashed black line correspond to observed mean agreement. Where the y axis is 0, this indicates line of perfect agreement.

Bland-Altman plots are presented for IPA and SI in Fig. 2, Fig. 3 respectively. In these plots, negative values on the y axis (that is, the difference in measurements between systems) indicate that the ViPerform recorded smaller values than the Delsys system, whereas positive values indicate that the ViPerform recorded greater values than the Delsys system. For IPA across both limbs, speeds and testing days, the mean difference was between −0.81 and −2.03 g, and for IPA SI was between −2.65 and −11.30%. For IPA and SI, the 95% LOA consistently extended beyond the respective 1 g and 10% thresholds set *a priori,* which indicated an unacceptable level of agreement.

## 4 Discussion

This study determined the within and between-session reliability of a research orientated and a clinically orientated IMU system, and the levels of agreement between these two systems for measurement of IPA and IPA SI during running in elite footballers.

Both systems demonstrated good to excellent within-session reliability for IPA and IPA SI across both speeds and testing days. However, the Delsys system had greater ICC values and less measurement error compared to the ViPerform system, possibly due to its higher sampling frequency. SEM values increased for both systems at 18kmph, which indicated that as well as increasing impact shock, faster speeds also reduced precision. Consequently, because of its superior precision, only the Delsys IMU had acceptable MDC values for within-session measurements of IPA at 14kmph, and SI at both 14 and 18kmph. This means that although both systems could be reliably used to evaluate IPA and IPA SI in one session, only the Delsys IMU could be used to determine the immediate effect of an intervention on running kinetic asymmetry, such as using a heel raise, change in footwear or alteration of running technique.

In terms of between-session reliability for IPA, both systems demonstrated good ICCs at 14kmph where values were 0.75 and 0.76 for the ViPerform system and 0.83 for the Delsys system. The Delsys values in our study were similar to ICCs previously observed at 1 week (ICC = 0.95) and 6 month (ICC = 0.94) measurement intervals, using an alternative research orientated system at comparable running speeds (Sheerin et al., 2018). However, we found that for IPA at 18kmph, ICCs generally deteriorated and were variable, accompanied by generally increased SEM values for both systems. Although IMU application was standardised, minor differences in placement between testing days could explain this increase in error. Also, day to day within-participant variability of impact loading patterns may also have contributed. Dependent on limb preference and speed, the consistently large between-session MDCs mean that only changes in IPA of greater than 14.01–31.95% for the ViPerform or 15.79–19.08% for the Delsys system could be considered a true change in performance, which is too great to be considered clinically useful. For both systems, between-session IPA SI reliability was inferior to IPA reliability and the large between-session SI MDCs for the ViPerform IMU (range = 26.71–30.99%) and for the Delsys IMU (range = 22.37–22.92%) also mean that both systems may not be able to detect clinically important changes in SI performance.

Overall, the between-session performance of both systems for IPA and IPA SI measurement suggests that caution would be required when interpreting results on a session to session basis. We therefore question whether using IMUs to measure lower extremity kinetics during treadmill running is a robust method of clinical assessment if used during PHE to establish to baseline functional performance for rehabilitation monitoring or to determine potential prognostic factors (predictors) for injuries in this population.

We found that in elite footballers, mean IPA increased at faster running speeds. This has been observed in other populations (Greenhalgh et al., 2012, Mercer and Chona, 2015). We also found that on average and irrespective of the system used, IPA was consistently elevated in the preferred kicking limb compared to the non-preferred limb at both speeds, which has not been previously reported. Unsurprisingly, these side to side differences were also reflected in IPA SI values, which ranged from 1.62 to 12.93%, depending on running speed and the IMU system used. This range is less than 31.7% previously reported for tibial shock asymmetry measured in uninjured runners (Zifchock et al., 2006) but could be explained by differences in SI calculation. We calculated SI according to limb kicking preference, whereas Zifchock et al.(2006) calculated SI according to right and left limbs. Additonally, because the kicking action in football places different demands on the support limb and kicking limb musculature (Brophy et al., 2007), specific musculoskeletal adaptations that have occurred as a result of training exposure may also partially explain the asymmetry observed in our study. Our findings suggest that establishing limb kicking preference is a vital component of running gait evaluation in elite players. Importantly, this also means that during rehabilitation, aiming to achieve symmetrical impact loading during running may be inappropriate in this population.

However, the true magnitude of asymmetry is unknown due to the lack of agreement between the IMU systems. For IPA measurements, the mean differences between systems varied between −0.81 and −2.03 g whereas for IPA SI values, it ranged between −2.65 and −11.30% depending on running speed and limb preference. For both IPA and IPA SI, the wide between-system LOA highlight unacceptable variability in agreement around these mean differences. Only in one of the eight IPA scenarios (i.e. at 14 kmph for the preferred leg - Fig. 2(c)) would adjusting by the mean difference bring the LOA within the ±1 g region of acceptability. This indicates that generally, using mean differences to adjust values provided by either system would not be an accurate transformation. The lack of agreement suggests that data measured from each system cannot be used interchangeably or act as a replacement for the other system. Therefore, in the absence of a gold standard method of tibial acceleration quantification, IPA measured using either IMU should only be considered as an arbitrary measure of absolute tibial shock, and IPA SI should be considered as an arbitrary measure of between limb tibial shock symmetry.

The lack of agreement between systems could be due to several reasons. Tibial IMU placement may have affected the magnitude of measured accelerations. Sensors placed at the distal tibia have been shown to register greater tibial accelerations that those sited at the proximal tibia when running at 8, 10 and 12 kmph (Lucas-Cuevas et al., 2017). Although the IMUs in our study were sited only 2 cm apart and the running speeds were greater compared to those investigated previously (Lucas-Cuevas et al., 2017), we also found that the distally sited Delsys IMUs consistently registered greater IPAs. Also, despite our attempts to maintain comparability between systems in terms of raw data filtering, the ViPerform system processed data using proprietary software algorithms which were unknown and potentially different to processing undertaken with the Delsys system. Finally, ViPerform IPA values were given to the nearest integer unit of gravity (i.e. as whole numbers) compared to the Delsys IMU, where values used two decimal places. This means that the ViPerform system was less sensitive to IPA changes.

### 4.1 Limitations and future research

This study has some limitations. Order bias was not controlled for as two chosen running speeds were evaluated sequentially. Although unlikely, this may offer an alternative explanation for the global increase in IPA measurements at 18kmph. Further studies could be conducted with a counterbalanced or randomised order and a wider array of running speeds. The generalisability of our findings is limited to elite football players only.

## 5 Conclusion

This study found that two IMU systems used to analyse treadmill running of elite footballers had good within-session reliability for IPA and IPA SI. Only the research orientated IMU could be considered useful to determine within-session changes of IPA at 14kmph and IPA SI at 14kmph and 18kmph, which has a restricted clinical application. Between-session analysis has shown a deterioration in reliability and poor MDC performances in both systems. The lack of agreement between the IMUs suggests that their data cannot be used interchangeably. Therefore, the use of IMUs to evaluate treadmill running kinetics cannot be recommended as a PHE test to identify prognostic factors for injuries or for rehabilitation purposes in this population. Further investigation of other IMU systems is required in elite football players and other populations to firmly establish their clinical usefulness.

## Data sharing statement

The participating football club granted permission to use the collected data for research purposes. An anonymised summary of the dataset generated and analysed during the current study may be available from the corresponding author on reasonable request.

## Funding

The lead researcher (TH) is receiving sponsorship from Manchester United Football Club to complete a postgraduate PhD study programme. This work was also supported by Arthritis Research UK: grant number 20380.

## Conflict of interest

None declared.

## Ethical approval

Informed consent was not required; all data were captured from mandatory PHE processes completed through the participant’s employment. The use of the data for the current purpose was approved by the Research Ethics Service at the University of Manchester.
